# Supplementary material for: Prioritizing bona fide bacterial small RNAs with machine learning classifiers
Source: PeerJ. 2019 Jan 24;7:e6304. doi: 10.7717/peerj.6304 (PMC6348098; doi:10.7717/peerj.6304)
Supplement: Supplemental Information 1 — Mean rank per model and PRC on five validation datasets. [file peerj-07-6304-s001.pdf]

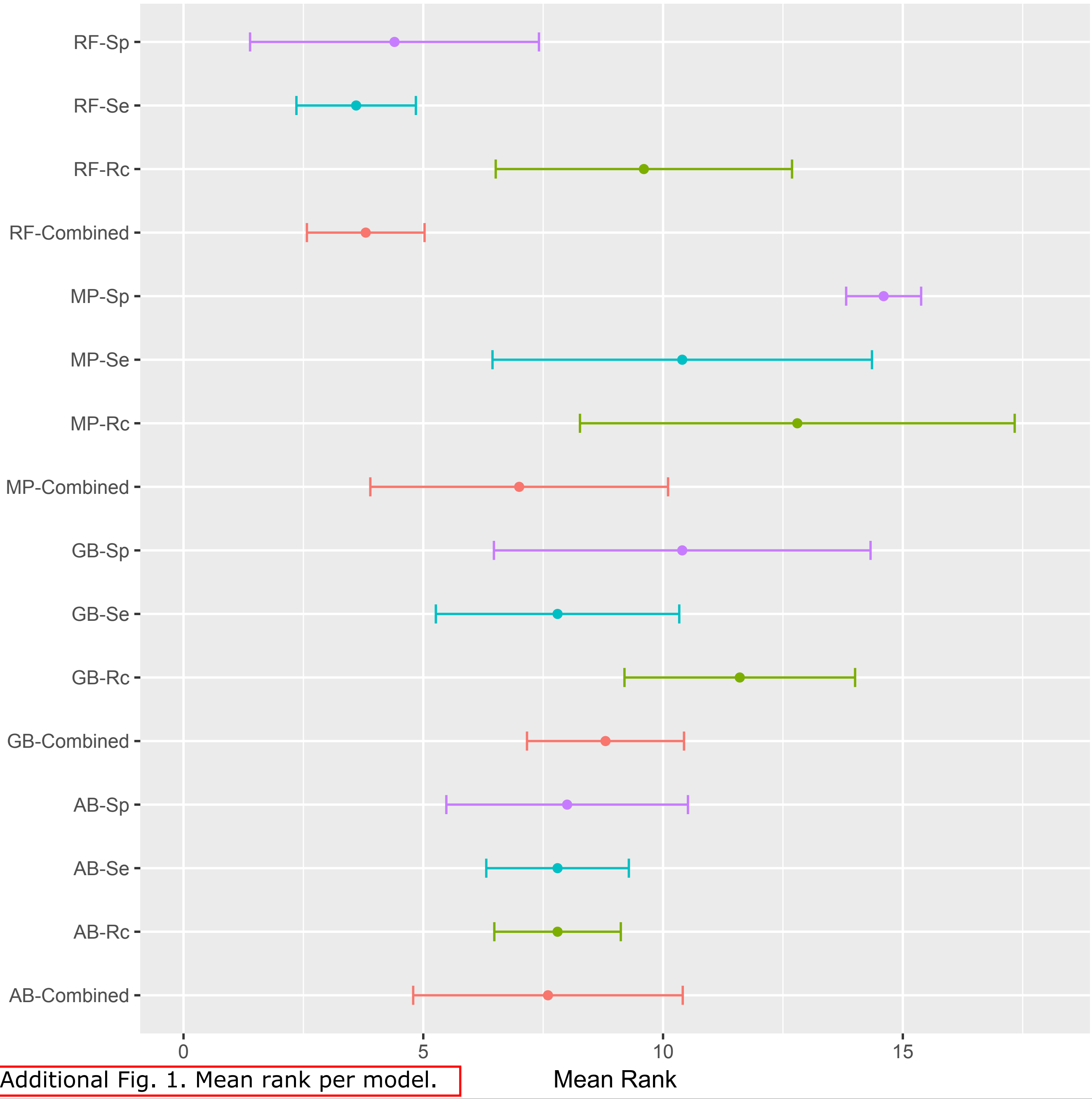

Additional Fig. 1. Mean rank per model.

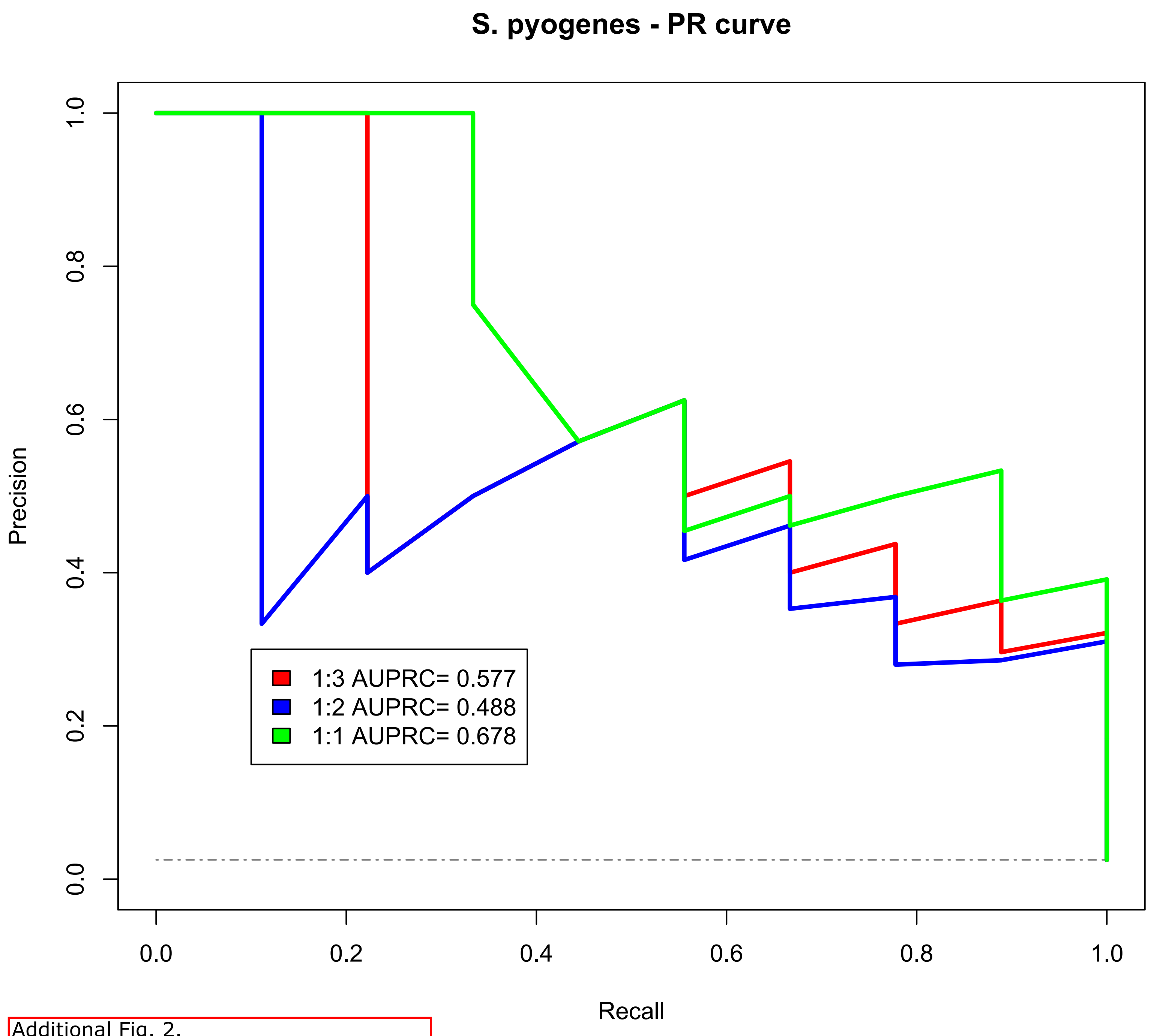

Additional Fig. 2.

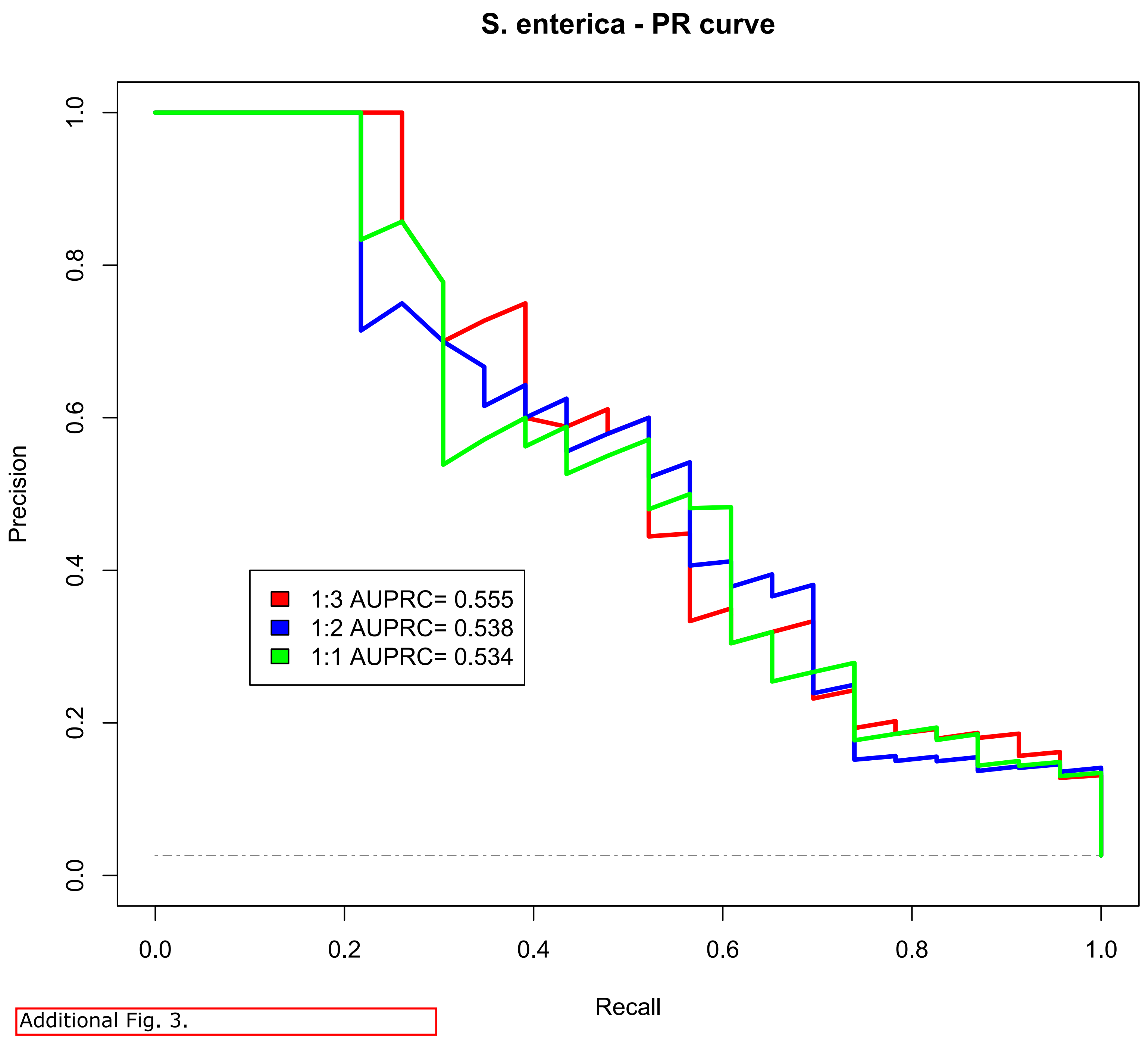

Additional Fig. 3.

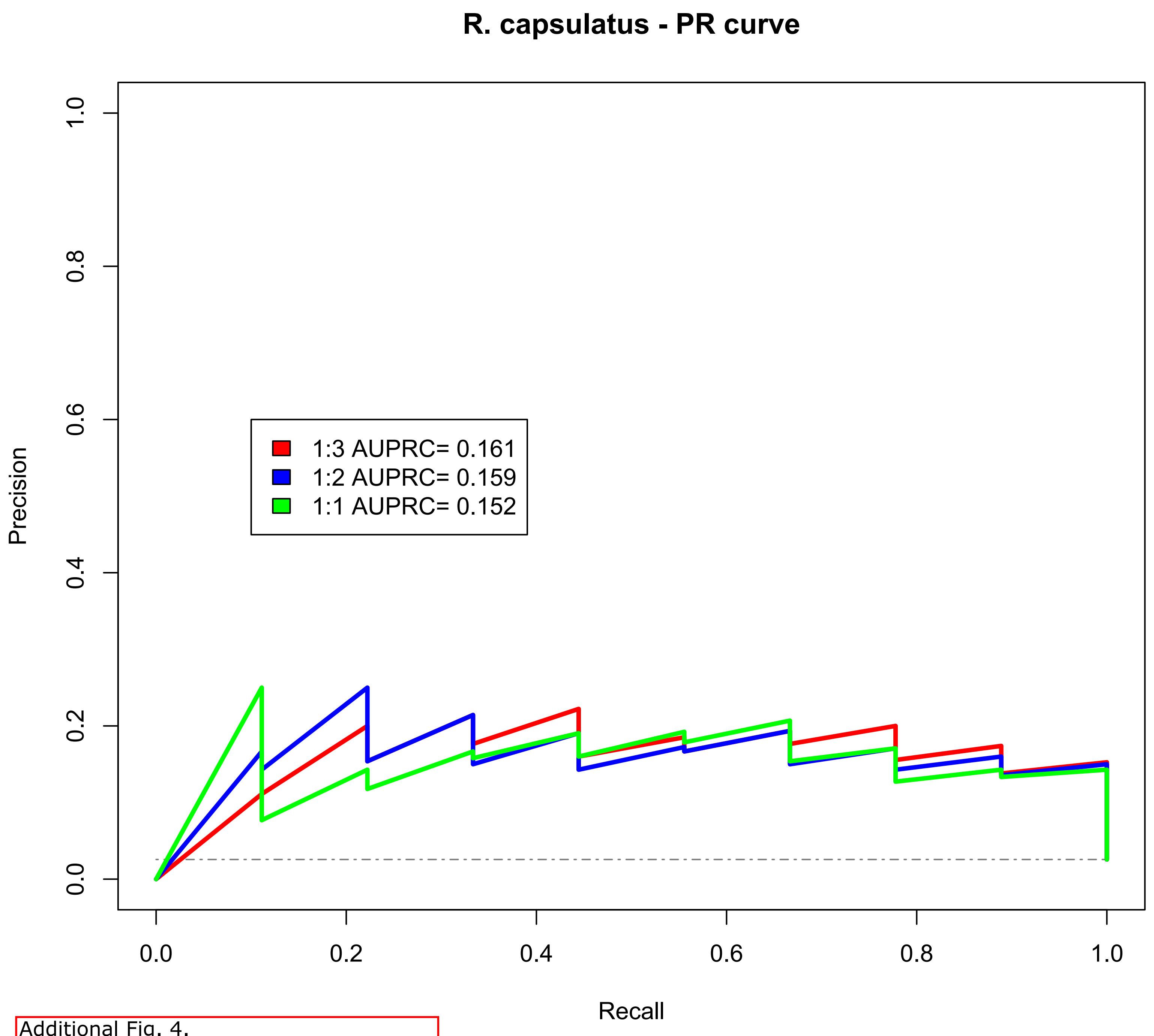

Additional Fig. 4.

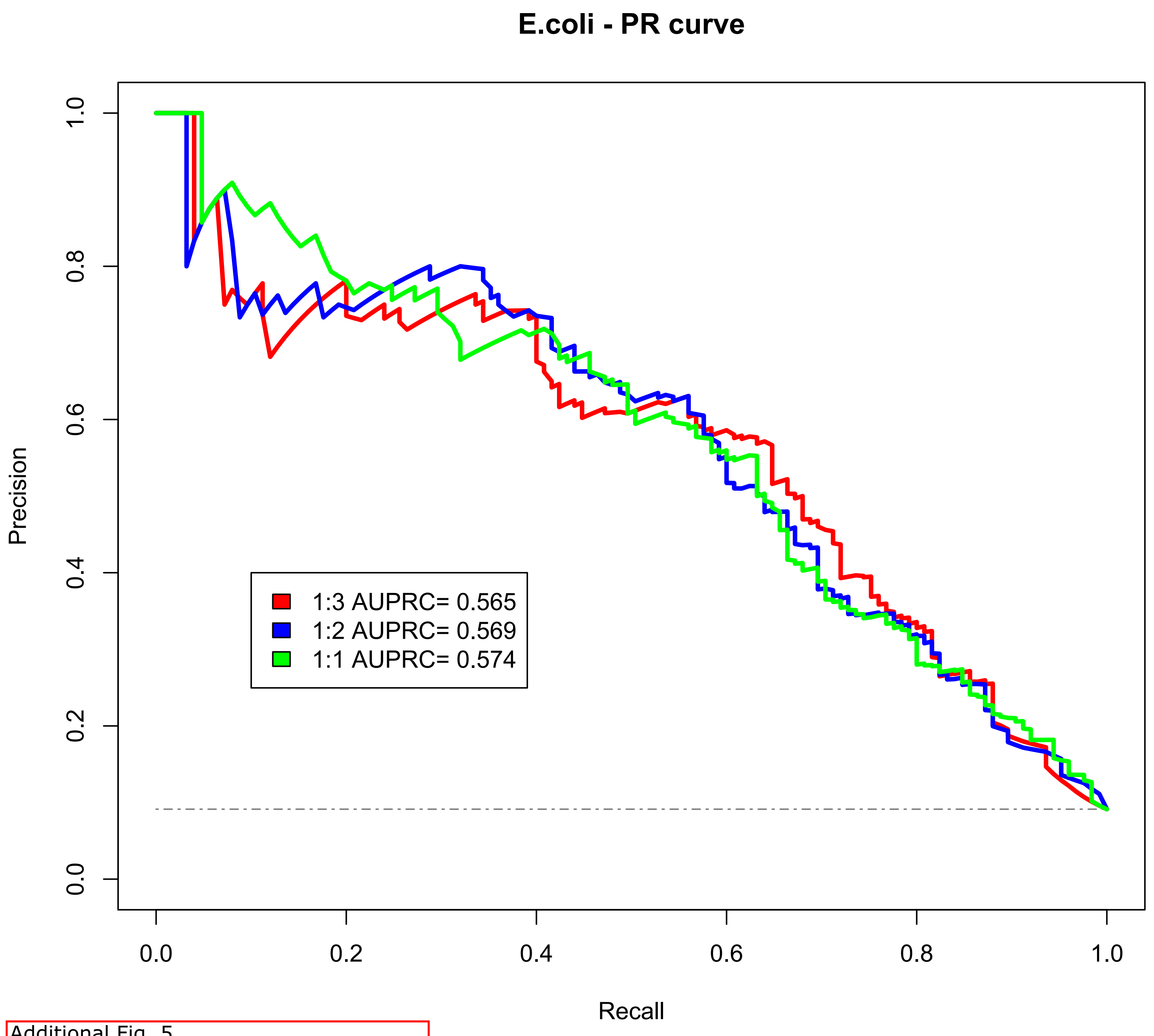

Additional Fig. 5.

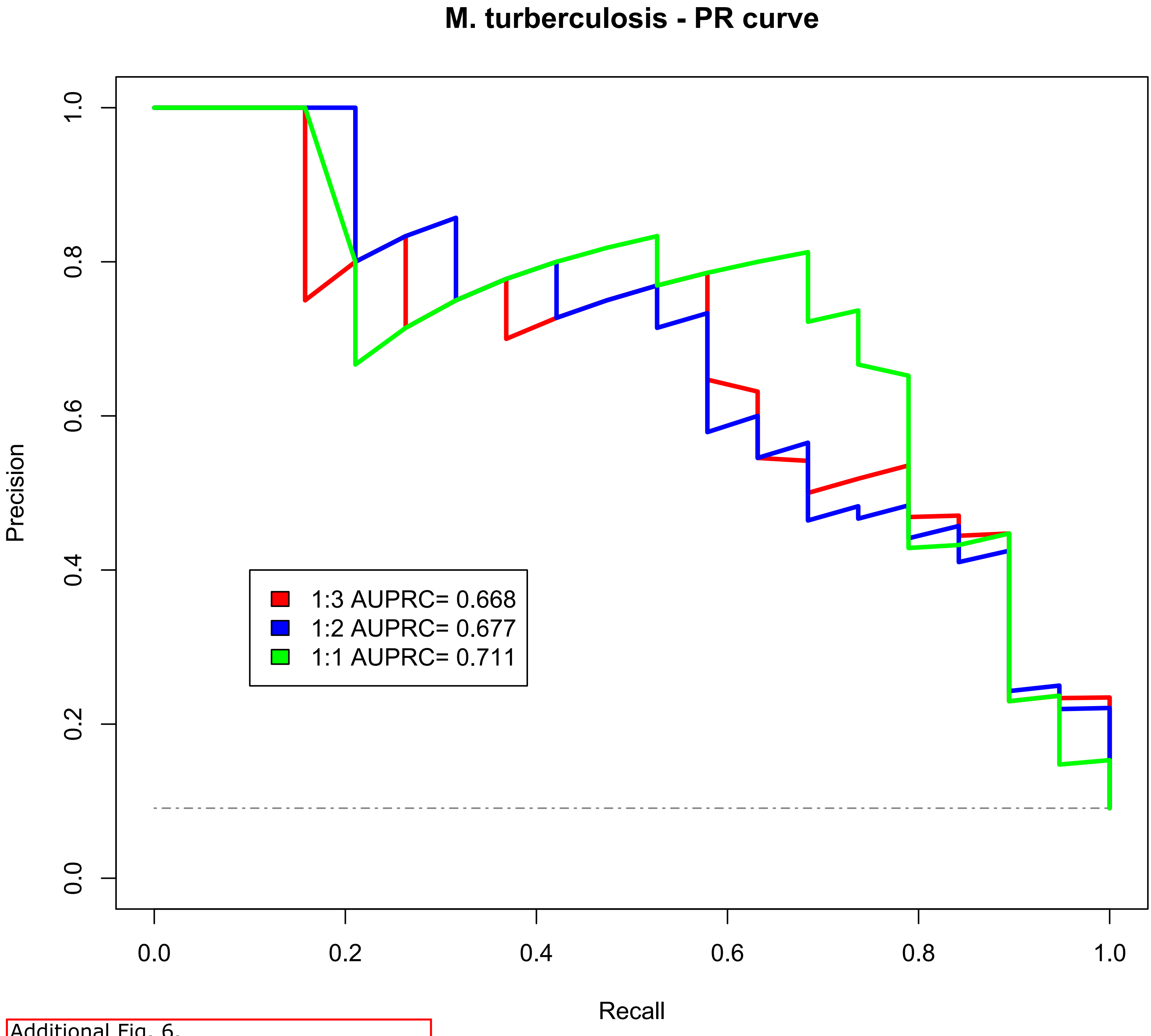

Additional Fig. 6.

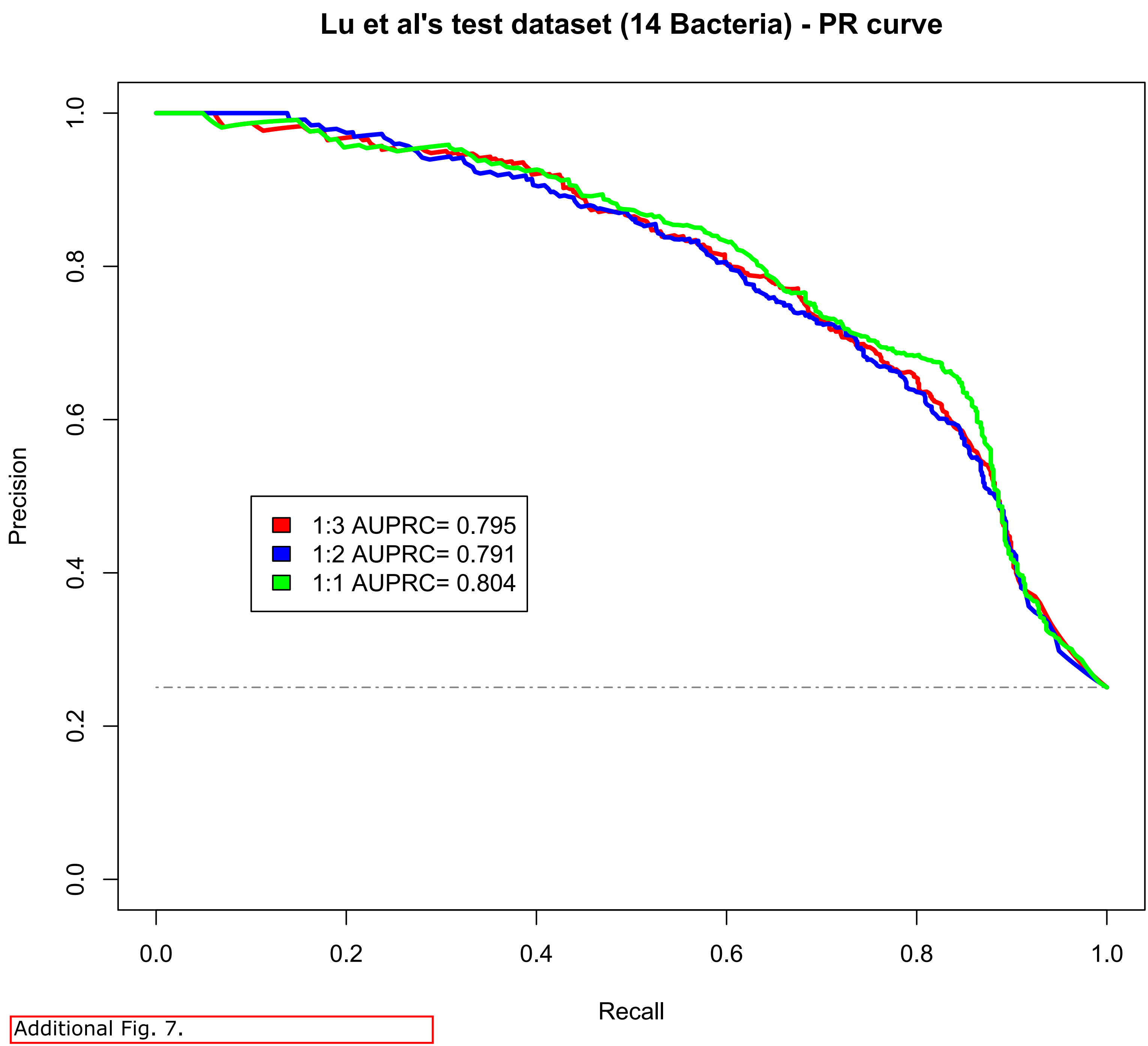

Additional Fig. 7.

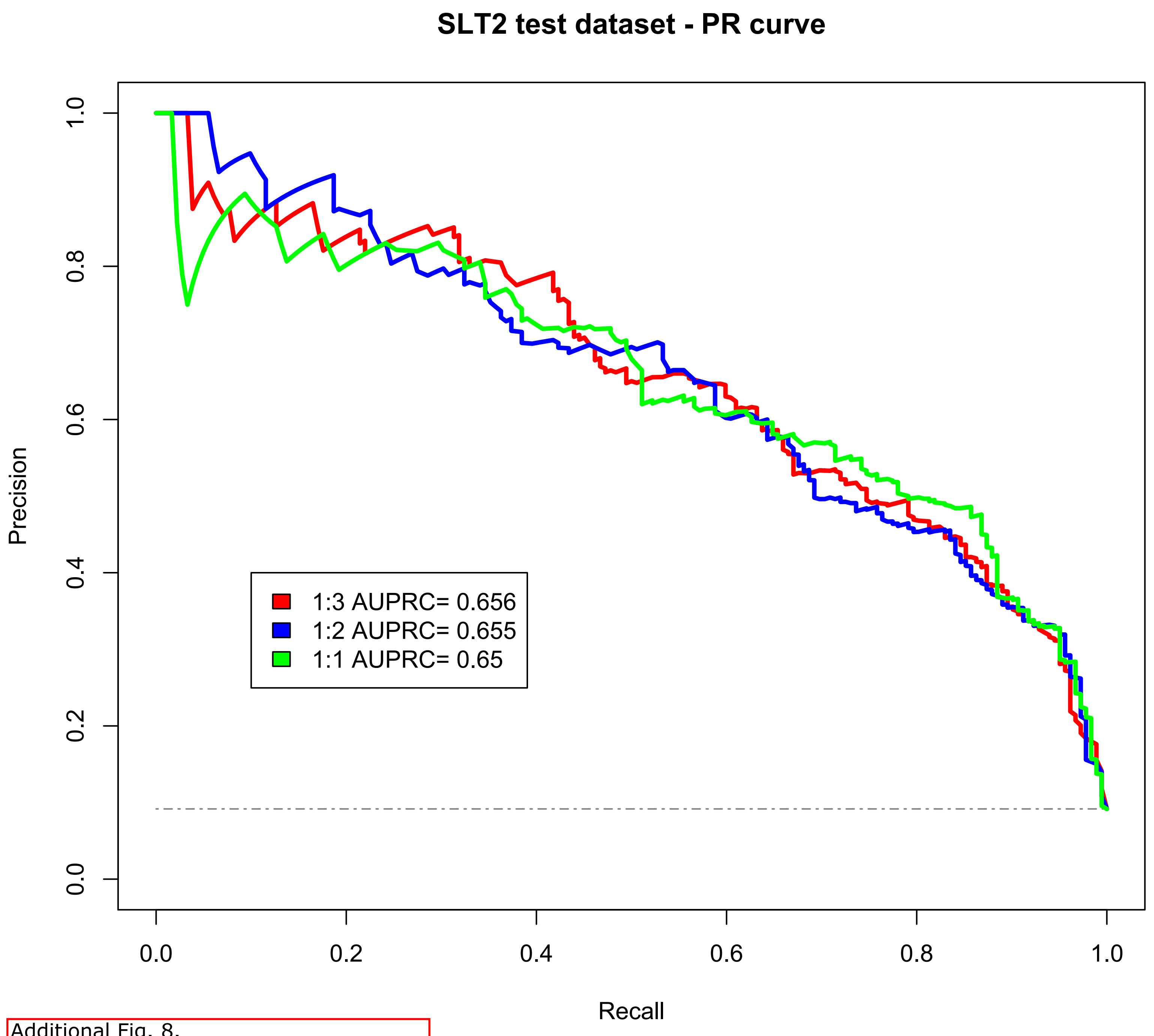

Additional Fig. 8.
